# Case Report: Optimal utilization of marginal lung allografts by considering donor–recipient PGD risk compatibility and by mitigating allograft and recipient inflammatory risk

**DOI:** 10.3389/frtra.2024.1450376

**Published:** 2024-10-03

**Authors:** Sue A. Braithwaite, Jitte Jennekens, Elize M. Berg, Linda M. de Heer, Faiz Ramjankhan, Michel de Jong, Jean Luc Charlier, Thomas C. Dessing, Marcel Veltkamp, Amy S. Scheren, Dieuwertje Ruigrok, Rob H. J. Schönwetter, Wolfgang F. F. A. Buhre, Niels P. van der Kaaij

**Affiliations:** ^1^ Department of Anesthesiology, University Medical Center Utrecht, Utrecht, Netherlands; ^2^ Department of Cardiothoracic Surgery, University Medical Center Utrecht, Utrecht, Netherlands; ^3^ Department of Pulmonology, University Medical Center Utrecht, Utrecht, Netherlands; ^4^ Heartbeat Perfusion, University Medical Center Utrecht, Utrecht, Netherlands; ^5^ Department of Pulmonology, St Antonius Hospital, Nieuwegein, Netherlands

**Keywords:** primary graft dysfunction, EVLP, cytokine adsorption, lung transplantation outcome, hypothermic oxygenated lung perfusion, lung transplant continuum, lung ischemia-reperfusion injury

## Abstract

Reducing the risk of high-grade primary graft dysfunction (PGD) is vital to achieve acceptable short- and long-term outcomes for recipients following lung transplantation. However, the utilization of injured lung allografts, which may confer a higher risk of PGD, must be considered due to the disparity between the increasing number of patients requiring lung transplantation and the limited donor pool. We describe a case in which highly marginal lung allografts were utilized with a good post-transplant outcome. Donor–recipient PGD risk compatibility was taken into consideration. Normothermic *ex vivo* lung perfusion (EVLP) was utilized to functionally assess the allografts. A second cold ischemia time following EVLP was avoided by converting the EVLP mode to a hypothermic oxygenated perfusion setup from which the lungs were transplanted directly. We attempted to mitigate lung ischemia-reperfusion injury in the recipient by employing cytokine adsorption both during the EVLP and intraoperatively during the implant procedure. In this case report, we describe our hypothermic oxygenated perfusion setup on EVLP for the first time. Furthermore, we describe the utilization of cytokine adsorption in two phases of the same transplant process.

## Introduction

Primary graft dysfunction (PGD) describes a phenomenon of acute lung allograft injury that occurs following lung transplantation. PGD is graded (ranging from low-grade 0 to high-grade 3) at four time points at 0, 24, 48, and 72 h after reperfusion of the second lung in the recipient according to the 2016 International Society for Heart and Lung Transplantation primary graft dysfunction definition (1). The outcome following lung transplantation is highly dependent on the occurrence and grade of PGD (2). There is no specific treatment for high-grade PGD: supportive care is best practice with consideration given to lung-protective ventilation strategies and, if necessary in high-grade PGD, the initiation of extracorporeal life support (ECLS) to facilitate lung rest (3). Prevention of PGD, where possible, is therefore paramount in any given lung transplant process.

Here, we describe a case where, initially, standard lung allografts, which at the time of procurement had become marginal lung allografts as identified by a partial pressure of oxygen (PaO_2_) in the arterial blood at an inspired oxygen fraction (FiO_2_) of 100% (P/F ratio) of <300 mmHg and an *en bloc* weight of 1,492 g, were safely utilized. Where possible, mitigation of the cumulative PGD risk was attempted throughout the transplant process. Donor–recipient PGD compatibility was considered. Furthermore, the prevention of additional, cumulative injury to the marginal lung allografts was undertaken with the use of a hypothermic oxygenated mode of *ex vivo* lung perfusion (EVLP) following a period of normothermic EVLP to prevent a second period of cold ischemia. We describe our hypothermic oxygenated EVLP preservation technique for the first time. In addition, lung ischemia-reperfusion injury (LIRI) in the marginal lung allografts was considered and risk reduction was attempted through the use of cytokine adsorption both during the EVLP phase and intraoperatively.

The outcome of the transplant process was as follows: the donor lungs had an out-of-body time of 13 h and 42 min and 16 h and 3 min for the right and left lungs, respectively. Following the transplant, the recipient was extubated after less than 2 days with a PGD grade of 1 at 72 h. The recipient was discharged home 26 days after the transplant.

## Case description

We received an offer of standard donor lungs for a recipient with a high risk of PGD from a donation following a brain death (DBD) procedure. The donor was a 55-year-old man, 185 cm tall and weighing 95 kg (BMI 28 kg/m^2^), who was admitted 2 days previous to the donation procedure with an intracerebral hemorrhage. He had no relevant medical history and no smoking history. The P/F ratio was 371 mmHg 6 h before the donation procedure and a chest CT scan showed a small band of atelectasis in both basal regions of the lungs. Echocardiography showed a left ventricle with concentric hypertrophy and normal contractile function with evidence of mild diastolic dysfunction and increased left ventricular filling pressures.

The recipient was a 50-year-old woman with interstitial lung disease and pulmonary arterial hypertension secondary to diffuse cutaneous systemic sclerosis with a lung allocation score (LAS) of 73. She had undergone an autologous stem cell transplant 4 years previously which had improved her cutaneous symptoms. At the time of the transplant, she had no skin complaints, dysphagia, or gastrointestinal reflux problems. In the prior month, the recipient had experienced an acute exacerbation of her interstitial lung disease that had been triggered by a respiratory viral infection. She had also experienced episodes of hemoptysis due to diffuse alveolar hemorrhage. Her pulmonary artery (PA) pressures were raised at 61/20 mmHg (mean of 39 mmHg) with good biventricular cardiac function. At the time of the offer, she was supported with high-flow nasal oxygen (Optiflow®) with 50% FiO_2_ at 50 L/min. Due to the habitus of the recipient [height of 162 cm, weight of 55 kg, BMI of 21 kg/m^2^, and a total lung capacity (TLC) range of 3.9–4.9 L] and the urgency of the transplant, she was accepted for eventual lobar transplantation depending on the TLC of the donor and, accordingly, as the TLC of this specific donor was 7.7 L, the plan was for a lobar transplant of the right middle and upper lobes and the left lower lobe (10 segments in total).

Immediately prior to the donation procedure, the P/F ratio in the donor had deteriorated to 270 mmHg and was not responding to recruitment maneuvers. Bronchoscopy was unremarkable. The lungs were procured semi-inflated *en bloc* according to standard practice and flushed in an antegrade direction with 4.5 L of Perfadex® Plus solution in the donor and again in a retrograde direction with 1.5 L of Perfadex® Plus solution on the back-table. On removal, both lungs were found to be abnormally heavy with an *en bloc* weight of 1,492 g (normal range 300–500 g without vascular perfusion) with no demarcated areas of atelectasis or other focal abnormalities. Differential diagnoses included neurogenic lung edema or edema due to left ventricular diastolic failure. Following discussions with the implanting surgeon and the associated team, it was decided that these edematous lungs were potentially unsuitable for lobar transplant in the designated recipient due to the high risk of PGD.

The lung allocation was given back to Eurotransplant, after which they were reallocated to a reserve recipient from our center with a lower disease severity who matched the TLC of the donor. The lungs were put on the EVLP with three possible outcomes: the lungs may further decrease in function and increase in weight, in which case they would not be suitable for transplantation; the extravascular lung water may resolve, the weight decrease, and the function increase, in which case they would be suitable for the original high-risk recipient; or, finally, the extravascular lung water may not resolve but not worsen and the function of the lungs increase, in which case they would be suitable for the reserve recipient.

The reserve recipient was a 66-year-old male, 193 cm tall and weighing 79 kg (BMI 21), with a pre-transplant diagnosis of combined pulmonary fibrosis and emphysema and an LAS of 33. His TLC range was 7.5–10 L. His past medical history showed floor-of-mouth cancer (cT2N0M0) which had been surgically resected 5 years previously. The patient was an ex-smoker with a 20 pack-year history. He was pre-operatively dependent on continuous nasal oxygen (2 L/min) and was ambulant and living at home, but, due to rapidly worsening dyspnea, had a minimal exercise tolerance. At the time of screening 3 months prior, he had slightly raised pulmonary artery pressure of 37/18 mmHg (mean of 26 mmHg) with no further irregularities in his cardiovascular screening.

### EVLP run and findings

The lung allografts were procured and transferred to our center in ice-based static cold storage with a cold ischemia time (CIT) until the start of the EVLP of 240 min. The EVLP was performed on an XPS® (XVIVO) system where the lungs were warmed to normothermia, perfused, and ventilated according to the Toronto protocol (4) with a modification of the ventilation parameters in which we avoided the hourly temporary increase in ventilation parameters as a recruitment maneuver but instead performed an hourly oxygenation test with an FiO_2_ of 100%.

A cytokine adsorber (Cytosorb® Cytosorbents) was built into the EVLP circuit. The inlet of the cytokine adsorber was connected directly to the outlet of the gas exchanger. The outlet of the cytokine adsorber was connected to the hardshell reservoir of the XVIVO Disposable Lung Circuit™. The flow of perfusate over the cytokine adsorber was started within 5 min after the start of the lung perfusion. A partial clamping of the PA tubing and an increase in the rotations per minute (RPM) of the centrifugal pump allowed for a passive flow through the cytokine adsorber of 400 ml/min. The PA pressure and the total flow to the lung were not affected by the circulation over the cytokine adsorber.

The difference in the PaO_2_ measured in a perfusate sample from the left atrium and a sample from the PA was 357 mmHg after 4 h of EVLP and the total weight of the lung allografts had increased by approximately 250 g (as measured by the continuous weight monitoring in the XPS® system). Our assessment was that the donor lungs were edematous due to diastolic failure in the donor which was not going to resolve after a standard time on the EVLP. The function of the lungs was satisfactory and, with adequate perioperative and postoperative management, we deemed the lungs to be acceptable for the reserve recipient.

After 4 h of normothermic EVLP, the lungs were cooled on the EVLP to 12°C. The ventilation of the lungs was stopped when the cooling started and the lungs were kept open with a continuous positive airway pressure (CPAP) of 10 cm H_2_O and an FiO_2_ of 40% and were perfused with a flow of 800 ml/min. The lungs were then transplanted one-for-one directly from this hypothermic oxygenated perfusion (HOPE) setup. The right lung was transplanted first according to our center's practice and was isolated on the EVLP by stapling the right main bronchus as proximally as possible and flush to the carina, utilizing an Endo GIA™ stapler (Medtronic). This ensured enough distance between the staple line on the right main bronchus and the right upper lobe bronchus for the ensuing anastomosis. The right pulmonary artery was ligated and the left atrium was opened. Following the removal of the right lung, the left lung remained inflated with a CPAP of 10 cm H_2_O on the hypothermic EVLP with a now-open left atrium.

**Figure 1:**
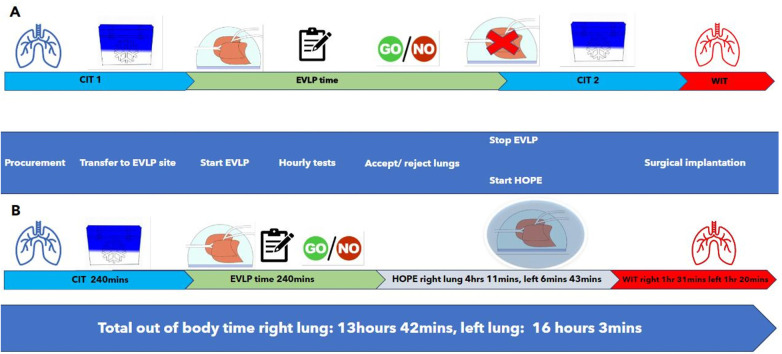
**(A)** A schematic diagram of the time intervals and events encountered in a standard DBD/EVLP procedure to contrast with **(B)** where the events and time intervals are given for a DBD/EVLP/hypothermic oxygenated perfusion procedure as was performed in this case. **(A)** The standard procedure of a static EVLP where a first cold ischemia time (CIT) is followed by a period of *ex vivo* lung perfusion (EVLP) to test and optimize lung function. This is then followed by a second period of static cold storage and cold ischemia (CIT 2) and a period of warm ischemia (WIT, warm ischemia time) during the surgical implantation in the recipient. **(B)** The time intervals and events in hypothermic oxygenated perfusion (HOPE) on the EVLP setup where CIT 2 is avoided. Actual times for the case are given in row B. During HOPE on EVLP, the lungs were cooled to 12°C, ventilation of the lungs was stopped, CPAP was applied in this case at 10 cmH_2_O, and the perfusion was reduced to 800 ml/min. The lungs were directly transplanted from the HOPE EVLP setup. In this case, the right lung was transplanted first, leaving the left lung on the HOPE EVLP setup with an open left atrium.

Both lungs underwent 4 h of normothermic EVLP with the right lung undergoing an additional 4 h 11 min and the left lung an additional 6 h 43 min of hypothermic EVLP. The use of the cytokine adsorber was stopped at the end of the hypothermic preservation. The events and their time intervals during the EVLP and hypothermic preservation phase are shown in Figure 1.

### Perioperative course

The transplant procedure was performed via a clamshell incision and veno-arterial (V-A) ECLS was instigated with cannulation of the right femoral vein, insertion of a multistage cannula to the level of the superior vena cava, and cannulation of the ascending aorta. V-A ECLS was used primarily to enable modified reperfusion of the donor lungs to minimize the risk of additional hydrostatic edema. A second Cytosorb was built into the ECLS circuit through which a flow of 500 ml/min was maintained during ECLS support.

Following removal from the EVLP, the right and left lungs were sequentially given an additional retrograde flush of approximately 2 L of Perfadex® Plus solution prior to surgical implantation. This is according to our local protocol. There were no signs of macroscopic thrombi in effluent from the retrograde flush.

After reperfusion of the second (left) donor lung, flow through the pulmonary circulation was kept low (approximately 2 L/min) for 45 min before the ECLS was weaned and stopped. During the reperfusion phase, as the patient was supported on V-A ECLS, the FiO_2_ of the lung allografts could be safely kept to 21%.

Following the closure of the patient’s chest, the lungs were ventilated with a positive end-expiratory pressure (PEEP) of 12 cm H_2_O, a driving pressure of 8 cm H_2_O, and a frequency of 14/min. This resulted in tidal volumes of 400 ml and, in the first arterial blood gas with an FiO_2_ of 100% following chest closure, a PaO_2_ of 445 mmHg and a PaCO_2_ of 58 mmHg.

**Figure 2:**
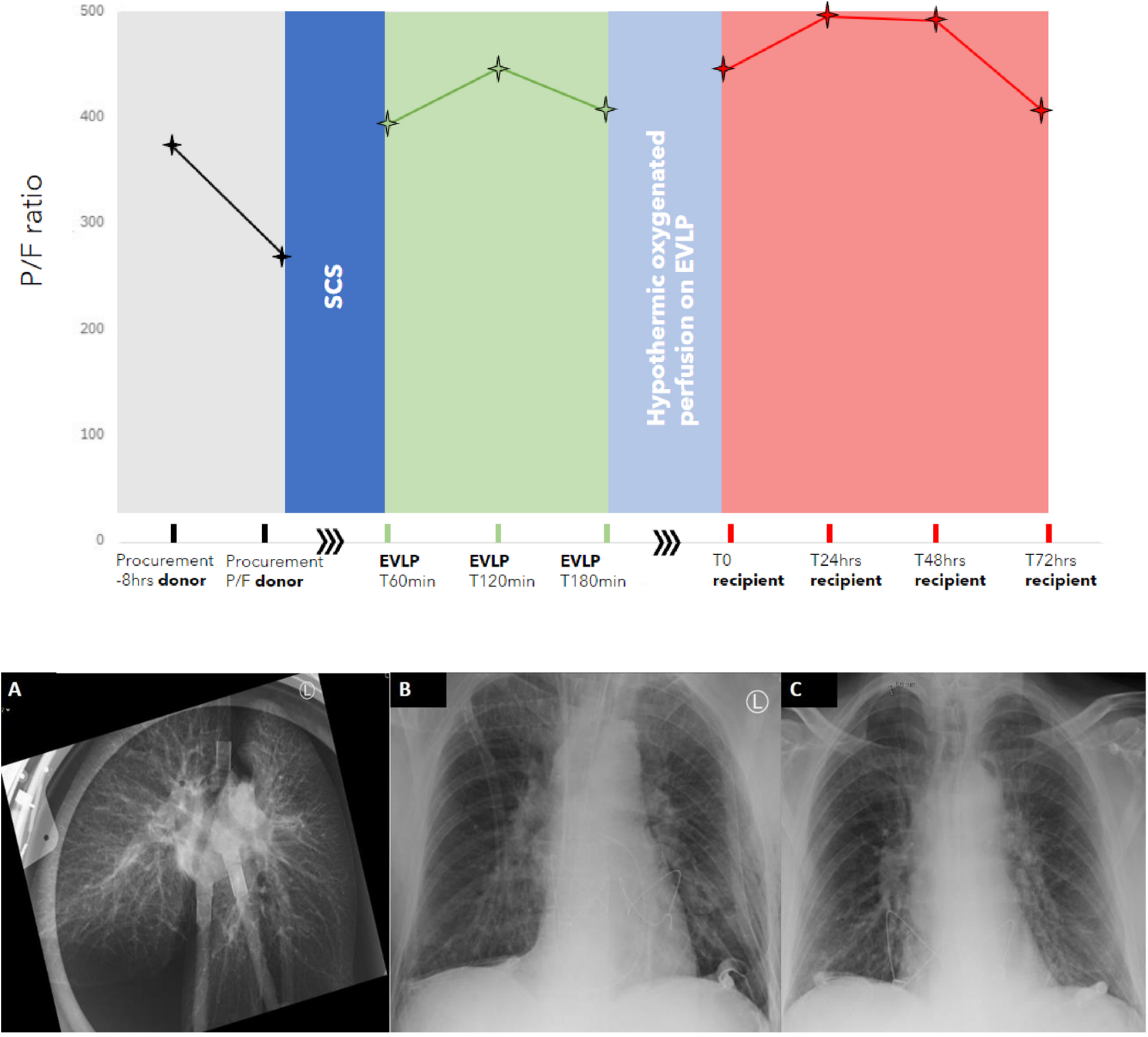
The PaO_2_/FiO_2_ ratio of the lung allografts during the transplant process from procurement in the donor to 72 h after reperfusion in the recipient. NB: The *x*-axis is not linear, the procurement and recipient phases are in hours, EVLP time is in minutes, and the post-transplant phase is in hours. SCS, static cold storage; EVLP, *ex vivo* lung perfusion. **(A)** X-ray at T120 mins of EVLP depicting a distribution of perfusate suggestive of hydrostatic lung edema. **(B)** Chest x-ray of the recipient on admission to the ICU immediately post-transplant. **(C)** Chest x-ray of the recipient at 72 h post-transplant.

The P/F ratios in the donor, during the EVLP, and postoperatively are shown in Figure 2 together with x-rays of the lung allografts during the EVLP and post-transplant.

### Postoperative course

The ventilation and oxygenation of the lung allografts remained stable in the postoperative phase with no evidence of lung ischemia-reperfusion injury. The patient was supported with noradrenaline for the first 24 h with a maximum dose of 104 ng/kg/min. No inotropics were required. Loop diuretics were started on postoperative day (POD) 2. The patient was extubated 42 h after ICU admission.

On POD 3, the PaO_2_ was 84 mmHg with the patient on room air (P/F ratio of 400) with mild edema on the chest x-ray (Figure 2) and a PGD grade of 1 at 72 h.

The patient was discharged from the ICU on POD 4. The rest of the hospital admission was complicated by subsegmental pulmonary emboli in the left lower lobe with no sign of secondary pulmonary hypertension (the CT PA was indicated by a limited period of breathlessness) and hospital-acquired pneumonia of the right upper lobe, treated with a 5-day course of antibiotics.

Kidney function remained within normal limits with the estimated glomerular filtration rate (as calculated by the Chronic Kidney Disease Epidemiology Collaboration formula in ml/min/1.73 m^2^) remaining above 90 for the first 20 postoperative days.

After a 26-day hospital admission, the patient was discharged home. The patient’s lung function post-transplant up until 92 days is shown in Table 1.

**Table 1:** Postoperative lung function and kidney function.

Days post LuTx	FEV1	Pred	% Pred	VC	Pred	% Pred	FEV1/FVC%	Pred	% Pred
32 days	4.34	4.00	109%	4.76	5.76	83%	91	75	121%
43 days	5.00	4.00	125%	5.42	5.76	94%	92	75	122%
57 days	5.35	4.00	134%	5.85	5.76	102%	91	75	121%
78 days	5.39	3.99	135%	6.17	5.76	107%	88	75	117%
92 days	5.50	3.99	138%	6.46	5.76	112%	85	75	113%

LuTx, lung transplantation; FEV1, forced expiratory volume in 1 s; Pred, predicted value; VC, vital capacity; FEV1/FVC, modified Tiffeneau–Pinelli index; proportion of the vital capacity able to be expired in the first second of forced expiration.

## Discussion

**Figure 3:**
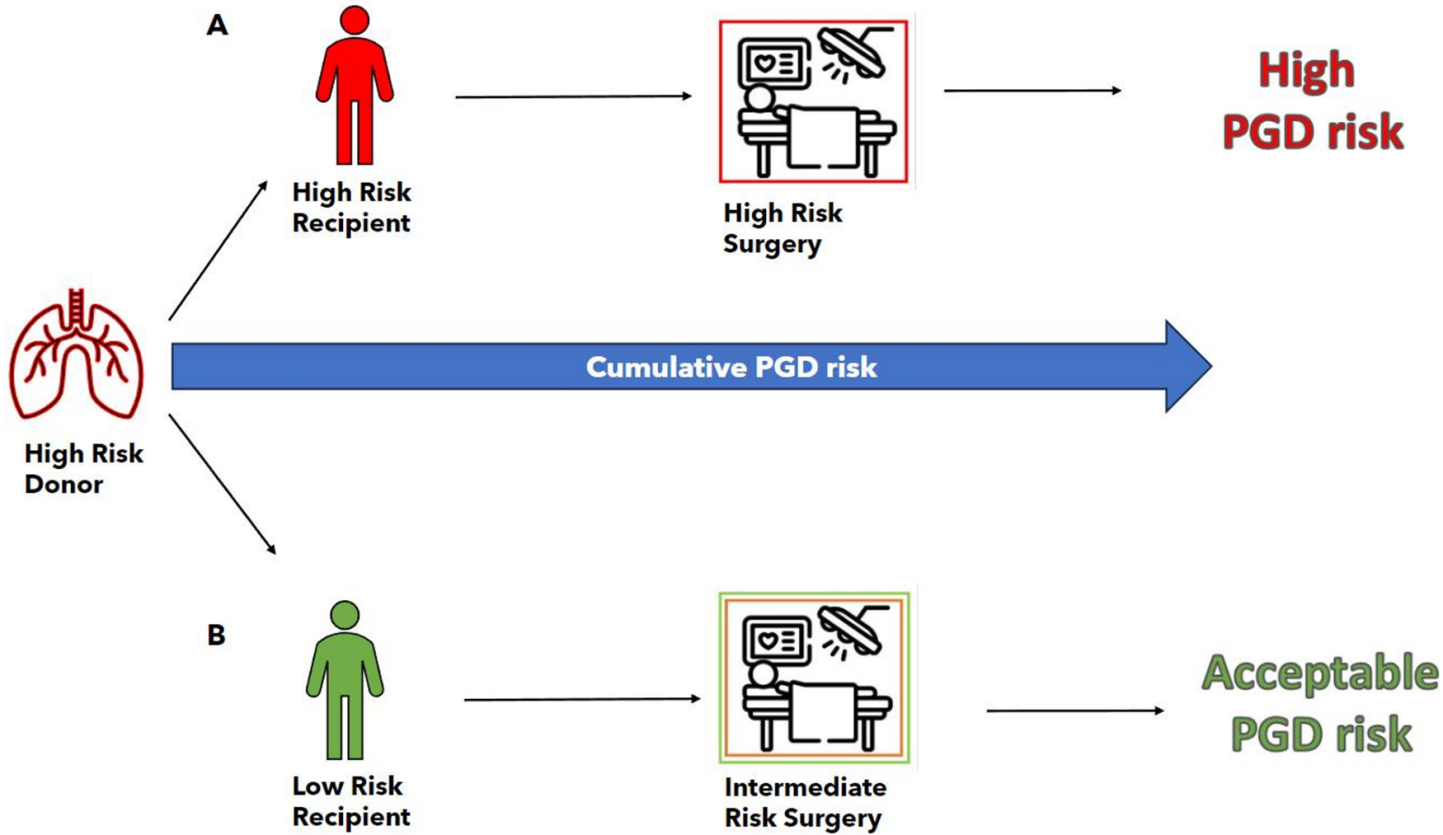
PGD risk estimation on the basis of recipient, donor, and surgical risk factors as taken in the context of a specific transplant continuum. The donor in this case is designated high risk due to the presence of severe edema (weight 1,492 g) and a P/F ratio of less than 300 mmHg. **(A)** Transplant continuum of the originally designated recipient with an estimation of a high cumulative PGD risk. The recipient was designated as “high risk” on the basis of (i) high lung allocation score (73), (ii) high-risk diagnosis (interstitial lung disease), (iii) high pre-operative disease severity (acutely worsening clinical state), and (iv) pulmonary hypertension (mean pulmonary artery pressure of 39 mmHg). Surgery was estimated as high risk due to (i) the necessity for size reduction of the graft in combination with (ii) the need for extracorporeal support during the operation. **(B)** The transplant continuum of the second (reserve) recipient with the estimation of the cumulative PGD risk as “acceptable.” The recipient was designated as “low risk” on the basis of (i) a low lung allocation score (33), (ii) a low-risk diagnosis (combined emphysema and fibrosis), and (iii) mild pulmonary hypertension (mean pulmonary artery pressure of 26 mmHg). Surgery was estimated as intermediate risk due to the need for extracorporeal support during the operation.

The risk of PGD in an individual recipient can be considered as the product of the cumulative risk incurred during a specific transplant donor–recipient-operative continuum. A clinical PGD predictive algorithm has been developed and validated by a group utilizing a prospective multicenter cohort study (the Lung Transplant Outcomes Group) (5). This algorithm (https://shiny.pmacs.upenn.edu/PGD_Calculator) utilizes center, donor, and recipient inputs to produce a predicted likelihood of PGD. While the donor risk inputs do not account for injured lung allografts in this case, the score gives an impression of the disparity in PGD risk between the two recipients in this report: the first high-risk potential recipient had a PGD risk score of approximately 75% and the second actual recipient had a PGD risk score of approximately 15%. The suitability for transplant of the donor lungs, as indicated by functional parameters and weight (as a proxy for extravascular lung water), was determined during the EVLP. In addition, the lung allografts were assessed in the context of PGD donor–recipient compatibility. As the weight of the lungs did not improve during the EVLP (the allografts gained 250 g) but the function (P/F ratio) increased, the lungs were deemed to be suitable for the recipient with the lower PGD risk profile (Figure 3).

We described our technique for HOPE on an EVLP circuit to avoid a second CIT in these injured donor lungs after a period of normothermic EVLP. According to our local protocol, cold ischemia refers to ice-based static cold storage. A second period of storage of lungs on ice has the potential to cause (further) damage to the lung endothelium at the mitochondrial level (6). Furthermore, a prolonged second cold ischemia time following an EVLP has been shown to be associated with increased PGD risk and 1-year mortality following a lung transplantation (7). In addition, hypothermic oxygenated perfusion has been shown in other solid organs to be a safe, feasible, and superior preservation mode compared to static cold storage (8–10). We have performed this technique in 15 pairs of donor lungs (data pending publication) with as yet no technical complications in the HOPE protocol. In all these cases, the lung allografts were assessed and accepted for transplant according to the Toronto Protocol on a normothermic EVLP, following which, the lungs were cooled and preserved in a HOPE setup as described in this case report. With regard to logistics, we perform all EVLP runs in an operating room (OR) directly next to the transplant OR, therefore the lung allografts can be transplanted directly one-for-one from the EVLP.

Due to the presence of extensive edema and a poor P/F ratio at the time of procurement, it was clear that the lung allografts in this case had incurred a perimortem injury in the donor. With the knowledge that any injury and ensuing inflammation in the donor lungs has the potential to become amplified upon reperfusion in the recipient (11), we attempted to mitigate any potential LIRI by incorporating a cytokine adsorber into the EVLP circuit. Cytokine adsorption has been used clinically in a case series in an EVLP setting where a reduction in inflammatory mediators in donor lungs was shown. However, the study had insufficient power to demonstrate a positive effect on outcomes (12). In a porcine damaged-lung allograft (ARDS) lung transplant model, cytokine adsorption helped restore lung function when instigated during organ preservation and continued post-transplant (13). It, therefore, seemed reasonable to continue the potential beneficial inflammatory modulatory effect of cytokine adsorption on LIRI during the intraoperative implant phase by incorporating a second cytokine adsorber in the perioperative ECLS circuit.

In this case report, we describe the safe utilization of marginal lung allografts by considering a number of factors that may have the potential to reduce the risk of high-grade PGD in a specific transplant process. This involved utilizing a normothermic EVLP to assess the lungs to consider donor–recipient PGD compatibility. Furthermore, a second, potentially detrimental, cold ischemia time was avoided for these injured allografts by converting the normothermic EVLP to hypothermic oxygenated perfusion for preservation. We attempted to mitigate any additional inflammatory risk of LIRI and PGD conferred by these marginal lungs by employing cytokine adsorption in two phases of the lung transplant process.

More research is required to be able to identify the specific lung allografts at risk of extensive LIRI that may benefit from perioperative cytokine adsorption. Furthermore, the EVLP-based HOPE for lung allografts protocol offers an alternative to ischemic static cold storage but requires comparative studies before routine clinical implementation.

## Patient perspective

Both recipients referred to in this case report have given written informed consent for the use of their medical details.

The first recipient was transplanted 11 days later than the allocation referred to in this case report. There was no PGD grade 3 in the first 72 h. However, her clinical course post-transplant was complicated by a severe critical illness neuropathy which necessitated protracted weaning from mechanical ventilation. She was discharged home after a hospital length-of-stay of 71 days post-transplant.

## Data Availability

The original contributions presented in the study are included in the article/Supplementary Material, further inquiries can be directed to the corresponding author.
